# Living With a Stigmatized Identity; Perceptions of Disclosure, Coping, and Medication Adherence Among Adolescent Boys and Young Men in Chiredzi-Zimbabwe

**DOI:** 10.3389/fpubh.2021.628725

**Published:** 2021-12-15

**Authors:** Roselyn Kanyemba, Kaymarlin Govender, Christopher Jimu

**Affiliations:** ^1^Health Economics and HIV and AIDS Research Division (HEARD), University of KwaZulu Natal, Durban, South Africa; ^2^Discipline of Psychology, University of KwaZulu Natal, Durban, South Africa

**Keywords:** stigma, disclosure, adherence, coping, adolescent boys, young men, ART

## Abstract

There is limited research on adolescent boys and young men (ABYM)'s initial and onward HIV seropositive status disclosure, coping strategies and treatment adherence journeys especially in Zimbabwe. This qualitative exploratory study employed in-depth individual interviews at Chiredzi General Hospital in Zimbabwe to explore the dynamics of disclosure, coping and treatment adherence among ABYM. Twenty-one HIV positive ABYM with ages ranging from 14 to 21 were recruited from their scheduled visit to collect medication at the hospital. Findings indicate that ABYM disclosure journeys began with shock, confusion or misunderstanding and ended in a positive life outlook. Treatment adherence among ABYM was very poor due to poverty, erratic food supply, feeling sick after taking medication, forgetfulness and the public nature of medication collection centers. The study concluded that ABYM maintained secrecy in order to be accepted by their peers but also to protect themselves from stigma and isolation.

## Introduction

With the widespread developments in improved access to and uptake of anti-retroviral therapy (ART), HIV has become easier to manage and has evolved into a chronic (rather than terminal) disease of childhood. However, HIV has proved not only to be a chronic disease but an extremely stigmatized one. HIV-related stigma has been identified as a significant stressor affecting Quality of Life of Youth Living with HIV/AIDS (YLWHA) (1). HIV/AIDS-related stigma is a complex concept that refers to “prejudice, discounting, discrediting and discrimination directed at persons perceived to have AIDS or HIV, as well as their partners, friends, families and communities” (2). Stigma can be: (i) enacted: overt discrimination based on HIV status, (ii) internalized: when a person absorbs negative messages or stereotypes about their HIV status and applies them to him/herself, or (iii) perceived: fear of being discriminated (2). Therefore, a stigmatized identity in adolescents affects how adolescence navigate their lives into adulthood through feelings of loneliness and marginalization. The quality of adolescents' future lives largely depends on how successfully they negotiate through this stage.

Adolescence as defined by the World Health Organization (WHO) is a developmental phase of physiological, cognitive and emotional development mainly characterized by sexual maturation and an increased desire for independence (3). Consequently, this developmental phase becomes an added complexity if an individual is living with a highly infectious and stigmatizing infection such as HIV (4). Adolescents' sexual experiences are similar, except that the experiences of HIV positive ABYM are “marred by anxiety over onward transmission, loss of freedom and spontaneity and a diminished sense of sexual attractiveness” (5). Studies have confirmed that majority of adolescents do not self-disclose their status for fear of rejection (6, 7). Instead, adolescents adopt different coping strategies to help moderate stress and these may include isolation, mental and behavioral disengagement, alcohol and drug abuse and adaptive strategies (e.g., seeking social support) among others (8). Studies have also confirmed how social stigma undermines prevention efforts by impeding HIV disclosure, preventative behavior and obstruct the uptake and adherence to treatment (9, 10). As such, studies emphasize the value of social support to support treatment uptake and adherence (11). Following this, it is favorable to consider the importance of ABYM's disclosure experience and journeys with regards to their seropositive status in a patriarchal society that exerts pressure on men to conform to socially valued representations such that those who fall short of these expectations are ridiculed.

There is a gap in research about the living experiences of ABYM living with HIV in terms of initial disclosure and onward disclosure as well as coping mechanisms and treatment adherence. Much of the research on adolescents living with HIV (ALHIV) in SSA has been confined to the experiences of female adolescents, particularly sex workers and young mothers, with considerable emphasis on social protection measures (cash transfers) to reduce path ways to HIV risk (12). As a result, issues and information about ABYM are poorly understood and documented in SSA. It is important to explore issues of disclosure in ABYM separately because grouping them together with females may mask particular gender disparities that may be useful in HIV interventions. Therefore, the broad objective of this study is to explore how HIV seropositive ABYM experience living with HIV and to examine their perceptions and experiences about disclosure and onward self-disclosure to friends, sexual partners, and others. Understanding the process of disclosure and its consequences for adolescents is critical for determining what types of support adolescents and parents need in order to disclose their status. This is in line with efforts toward closing the adolescents' treatment gaps in order to realize the 95-95-95 target by 2030 which will ensure that those children and young people who are infected and affected by the HIV/AIDS epidemic receive the prevention, care, and treatment services they need to survive and thrive. The study is guided seeks to answer the following questions;

- What are the barriers to HIV disclosure in ABYM?
- How does disclosure promote/inhibit an adjustment to living with HIV?

## Conceptual Framework

The study is grounded on the Social Ecological Model (SEM) by Bronfenbrenner (13). The theory acknowledges the intertwined relationship existing between an individual and their environment. The theory is especially useful for this study because it recognizes external factors that shape adolescent disclosure, coping and treatment adherence. In Zimbabwe there is a saying in Shona, “Munhu vanhu” (upon literary translation this says “a person is people.” This means that a person can only become a mature responsible citizen with support of other people in his/her family and community, Pg 88) (12, 14). Therefore, the reality of ABYM living with HIV is determined by the environment in which he interacts with which give meaning and direction to an adolescent's life.

We used qualitative methods and a social ecological framework to gain in-depth understanding of perceived barriers to disclosure as well as treatment and adherence (i.e., individual, micro-system, meso-system, exo-system, macro-system, and crono-system.) associated with living with a stigmatized identity. Each environmental context has effect on the development of the individual, in this case ABYM living with HIV. Self-stigmatization describes the “individual” level where stigma and discrimination exist as internalized structures based on misconceptions about the self, affecting ABYM's wellbeing, the “microsystem” is the context in which ABYM is situated and includes the ABYM's family, home, peer group, and school that affect their development and affect decisions on disclosure and consequent health choices such as accessing and utilizing healthcare services. This suggests that more nurturing and supportive interactions and relationships will likely to foster a better environment for development. The next level is the “mesosystem” which includes the interactions among two or more microsystems which can provide much of the context for socio-emotional development. This includes the relationships between ABYM's peer group and family which should typically be supportive of ABYM disclosure and living experiences. The layer beyond the mesosystem is the “exosystem,” which contains two or more settings; however, only one interacts with the individual. Exosystems are typically organizations that includes health services, such as hospitals and support clinics that facilitates medical and emotional support toward disclosure and acceptance of a seropositive status thereby having great impact on initial and onward disclosure and health-seeking behaviors of ABYM. The outer layer, the ‘macrosystem' encompasses the broader societal and cultural norms and socio-economic influences on development of ABYM. Examples of factors within the macrosystem include customs, knowledge systems and cultural beliefs that usually guide ABYM's behaviors. In the case of ABYM, these might include hegemonic masculinity scripts that discourage males from accessing healthcare systems and taking medication but rather encourages multiple sexual partnership and displays of physical strength as being representative of a true man. Bronfenbrenner (15) referred to the macro system as the “blueprint” for social structures. Each of these ecological systems inevitably interact with and influence each other in all aspects of the children's lives.

### Justification for the Study

In sub-Saharan Africa, the main mode of HIV transmission is through heterosexual sex with a concomitant epidemic in children through vertical transmission. Of the global population of adolescents living with perinatally acquired HIV (ALPHIV), 79% live in sub-Saharan Africa (SSA) (16) where AIDS is a leading cause of death for adolescents (17, 18). Between 2000 and 2015, annual AIDS-related deaths declined in SSA for all age groups except adolescents (aged 10–19 years), where mortality more than doubled from 18,000 to 41,000 (17, 18) This is in part due to non-uptake of prevention, treatment and care opportunities due to the stigma attached to the disease. Therefore, understanding experiences of ABYM is critical in exploring a biomedical holistic intervention that must be health promoting and focusing on reducing onward transmission. Findings from this study will contribute to the already existing field of knowledge and contributes to policy formulation and practice aimed in reducing new infections in Zimbabwe. Findings will also assist ALHIV in the promotion of health, therapeutic compliance, safer sex and recovery from illness (19).

### Study Design, Tools, and Sampling Procedure

A qualitative exploratory design guided this study from October to December 2019. The study employed purposive sampling to select 21 ABYM who are HIV seropositive and know their status with ages ranging from 14 to 21. Initially, 24 participants had accepted to participate in the study but 3 pulled out because they were no longer interested in the study.

Participants were recruited while accessing HIV social support services at Chiredzi General Hospital. Minors were accompanied by their parents and caregivers while the majors collected their medication themselves. All interviews (lasting for 45 min to 1 h) were conducted from October to December 2019. Initial access to the participants was negotiated through the hospital superintendent who then introduced the researchers [principal researcher who is a professor in psychology and two assistant researchers (one female with a PhD in anthropology and one male with an honors degree in psychology and counseling respectively)] to the participants. This was important to establish a good rapport with the participants. Potential participants were informed about the study and those who were aged 18 years and above voluntarily consented on their own. For those below the age of 18 consent was provided by parents or caregivers and they signed assent forms themselves. They were informed that they were going to participate in a face-to-face individual in-depth interview in the presence of either a psychologist or counselor to provide counseling in case they exhibited elements of psychological trauma. To reduce participant anxiety and fear of accidental disclosure to the community, the interviews were held in a secure space (the clinic's counseling rooms) and pseudonyms were used instead of their real names. The pseudonyms were derived from common Zimbabwean names. The interviews followed a semi-structured interview guide which covered issues of disclosure, coping mechanisms and treatment adherence. The semi-structured interview design was appropriate for this study as it permitted a focused exploration of a specific topic (20). Questions focused on ABYM's narratives of experiences of living with HIV and included the adolescents' biography information, experiences of disclosure of their HIV status, experiences of disclosure to others, experiences of disease management as well as traumatic experiences related to their HIV status. Questions also focused on ABYM's relationship with their families, peer groups, cultural expectations and health-systems and how they intersect in their day to day lives. Repeat interviews were conducted with five participants to clarify on issues that were not clear in their initial stage interviews. Field notes were made during all the interviews. Participation was voluntary with no direct benefits, but transport costs were reimbursed to the participants. Each participant was offered Zimbabwean Real-Time Gross Settlement (RTGS) money equivalent to R40 per day as transport costs. Recording permission was sought verbally and through informed consent forms. All interviews were audio recorded with the consent of the participants.

### Trustworthiness

We followed Lincoln and Guba's criteria for credibility, transferability, dependability and confirmability to enhance the trustworthiness of our study (21). All researchers involved in data collection completed GCP training. Researchers held several debriefing meetings during data collection and analysis of interviews and data saturation was reached. Transcripts were shared amongst the researchers to check for quality and to agree on coding and formulation of themes. Disagreements were discussed and common consensus reached. Data analysis and involved reading and re-reading verbatim transcripts of all interviews conducted, listing all topics, coding and categorizing themes. Recordings were transcribed, supplemented by written notes, and translated into English by one of the data collectors with master's level qualification in public health. Findings were coded and analyzed using NVivo software. Major themes related to disclosure were explored and presented as narrative. While our analysis prioritized, important themes based on how often they were raised by participants, the full spectrum of experiences, including divergent accounts were also considered. Adherence with the Consolidated Criteria for Reporting Qualitative (COREQ) research was determined by all three researchers (22). This was to enhance quality, rigor and credibility for this qualitative study.

In order to obtain rich data required to understand adolescents' experiences of living with HIV, a pilot test of the interview guide was conducted with five (*n* = 5) participants before the main study was done. Adolescents who participated in the pilot study were not part of the 21 participants that participated in this study, but they had similar characteristics. Piloting was done on a different day before the main study and modifications to the interview schedule were done 2 weeks prior to the interviews.

## Ethical Considerations

Ethical clearance was granted by the Medical Research Council of Zimbabwe approval number (MRCZ/B1794) and University of KwaZulu Natal BREC approval number (BE475/19).

## Findings

### Description of Study Participants

21 ABYM with ages ranging from 14 to 21 years participated in the study. 13 were aged 18 and below and 8 were aged 19–21 years (see Table 1). The mean age for participation was 17, 8 years. A third (*n* = 7) of ABYM had both parents as primary caregivers with the rest having grandparents, one parent or extended family as their primary caregivers. Perinatal infection was high with 15 of the 21 ABYM perinatally infected, one through suspected sexual intercourse, two were not sure where they acquired the virus and one suspected sharps such as razor blades and needles since they shared these at home and two did not know where they got the disease. Clinical disclosure was high with 14 ABYM having their status disclosed to them through a counselor, three were told by their mothers, one had their status disclosed by an extended family member and one had their status revealed by both parents. Initial disclosure occurred at a variety of ages ranging from 5 to 20 years of age with the mean age of initial disclosure being 19 years of age. All of the participants have been on ART for more than a year with the average age for taking ART being 5 years duration. The prevalence of self-disclosure to others was very low. Two had disclosed to their teacher, four to their friends, four to family members and three to their peers, and eight had not disclosed to anyone. ART adherence was low with one boy taking medication religiously, 19 reported that they sometimes forgot to take their medication and one reported that he had stopped taking medication completely although he still reported to the clinic for collection and social support.

**Table 1:** Profile of ABYM age, initial disclosure, self-disclosure, and ART duration.

	**Number**	**Percentage (%)**
Age of participant		
14–18	13	62
>18–21	8	38
Type of caregiver		
Grandmother	2	10
Grandfather	1	5
Both grandparents	2	10
Both parents	7	33
Father only	2	10
Mother only	3	14
Independent	2	10
Aunt	1	5
Stepmother	1	5
Mode of transmission		
Perinatal	15	71
Suspected sexual intercourse	1	5
Suspected sharps	1	5
Not sure	2	10
No idea	2	10
Age at disclosure		
5–10 years	8	38
>10–15 years	11	52
>15–20 years	2	10
Duration taking ART		
0–3 years	5	24
>3–6 years	6	29
>6–9 years	4	19
>9–12 years	6	29
Who initially disclosed to them		
Counselor	14	67
Father	1	5
Mother	3	14
Other	3	14
ABYM self-disclosure to		
Teacher	2	10
Friend	4	19
Family	4	19
Classmate	3	14
None	8	38
Level of ART adherence		
Always	1	5
Sometimes	19	90
Stopped voluntarily	1	5

### Themes Emerging From the Study

Disclosure for most of the adolescents was preceded by episodes of serious illness. When asked about the circumstances leading to the disclosure of their HIV status the majority of respondents (*n* = 14) reported they always felt sick before they knew about their status with the rest getting to know about their status after random testing.

*I was always sick when I decided to go to the clinic for testing. I was just connecting what I was learning at school and what I was feeling. I was 14 years of age then. (Taurai-21 years)*

*My parent were just giving me tablets. I did not know what there were for. I would just take them without question. When I was 12 years of age I was now coming alone to take pills. I later discovered through attending social support meeting that the tablets l was taking and the support group l was attending was for HIV positive people. (Vengai, 14)*

For some, disclosure was accidental. Terrence (15 years) had this to say:

*I went with my grandmother to collect my medication. I did not know what it was for. Then l was 10 years old. She had gone to the toilet and it was our turn to collect and she was still not back. I proceeded without her and told the nurse that l was collecting headache medication. I was holding this green card where they recorded collection dates. She then told me that the green card was for HIV medication not headache. That's when l knew*.

The discussion reveals how caregivers preferred the deception tactic whereby they ascribed the adolescent's health condition to a different illness or complete non-disclosure where there is complete secrecy around the diagnosis and the adolescent are not told the truth about their illness. A probable explanation may be because caregivers felt disclosing the truth will be too much a burden on minors and expecting them to take charge of taking their medication will be giving the minors a responsibility beyond their age. Withholding information from the minors on what the medication they were taking was for reflects elements of patriarchal culture in Zimbabwe. Culture emphasizes not questioning elders on their decisions as a sign of respect (23, 24). Therefore, caregivers borrowed this power to enforce compliance and maintain the secrecy of taking HIV medication. However, this might have negative effects as the children will not know the importance of taking medication correctly. In addition, the fact that caregivers chose not to disclose ABYM seropositive status reflects the importance of having an adult to enforce discipline in taking ART to prevent defaulting.

#### Perceptions of ABYM Disclosure-Shaky Beginnings

ABYM were asked how they received news of their seropositive status. All participants reported both positive and negative effects of disclosure. The prevalent initial reaction was anger followed by pain, confusion, and disbelief and some blamed their parents for the disease. While to some it was temporary, for others the period of shock was long-lasting. The majority of respondent (*n* = 10) reported feelings of anger, disbelief and stress and isolated themselves for varied periods of time with four feeling suicidal, three accepted their status without question because they already suspected they had HIV and 4 did not know how to react because they were too young to understand about HIV. It was noted that the helplessness and trauma emerged from the common belief that HIV is a disease synonymous with promiscuity and death. Takunda 14 years said;

*The day they told me l had HIV I felt bad, very bad, I thought so this is the end of my life and that I would never make my dreams come true. What did l do to get this disease yet l have never had sex. I was scared everyone would find out I am HIV-positive and would reject me…even now l am not sure if they don't see that l have HIV. I don't know what my future holds, I can die at any moment, my father died even though he took his medication religiously*.

Taurai 21 years old said;

*It was hard for me to accept. I even isolated myself for a month from others. I even thought of suicide, then l thought of my mother. How would she cope with me gone…? To some extent l blame my parents because l got it from them*.

These responses reflect the negative psychological potential of a seropositive status disclosure to minors. This underscores the need for social support both at home and clinics to support patients. Once diagnosed HIV-positive, people battle intense mental suffering, accompanied by anguish and fear as well as sadness caused by uncertainties regarding the future and reduction in self-esteem (11). Sometimes the feeling of guilt and blame marked by the prejudice that usually accompanies the history of the disease alters one's identity forever. However, a firm support structure usually strengthens and gives hope for the future.

To emphasize the importance of social support Tatenda (12 years) and James (13 years) took it in their stride because they received adequate counseling and support.

*At the hospital they told me everything, I never thought of committing suicide because I was counseled here at the hospital. Even at home my other two sisters take ARV's and l have been taking them since l was five. It was never a problem for me when they told me the pills were for HIV*.

*I was happy that they had finally told me. My mother would instruct me to take my tablets at a particular time of the day but they told me they were for asthma. But l was suspicious because l didn't have an inhaler like my friend who had asthma. Telling me about my status cleared the worries l had*.

This reflects evidence from studies of social and emotional development in adolescence, where support from families or other caring adults has been found essential for a range of healthy behaviors (25).

#### Coping With Stigma

Although the majority of adolescents were shocked and angered by their status, they quickly adapted to the situation some through maintaining complete secrecy, some disclosing to immediate family and some to a few trusted people. The majority (*n* = 8) did not disclose to anyone. They felt that if they revealed their status to peers and teachers, they risked getting rejected, isolated, and stigmatized.

*I have never told my status to anyone. I didn't even disclose my status to my girlfriend. I fear that if I tell her my status she will reject me and would continue to go and spread my status to other people. This is what I still fear. (Vengai, 19 years)*

*Some people will go and gossip about you. The will go and tell everyone at school and in the community. It once happened once when l was still at primary school, I disclosed to my friend and we quarreled then he went around telling other people. They began to avoid me and didn't want to sit close to me in class laughing at me. I reported them to my teacher who rebuked them. It only stopped for a while and they began again. (Tshiamo, 18 years)*

ABYM were asked about their experiences with living with HIV and experiences of stigma varied among participants. With regards to family disclosure participants had this to say;

*My close relatives are the ones stigmatizing me. They separate my food, soap and utensils. If they buy their cookies or snacks, they don't want to mix theirs with mine. Mine are kept in a separate cupboard. Even soap I use my own. It worries me a lot. Even blankets they don't want to share with me. (Diva, 19)*

*My mom helps me to keep the secret. My siblings do not know l have HIV, she calls me on the side to administer my medication. There is no need to tell my siblings because they will think we are different. (Jojo, 15 years)*

*My siblings know. Sometimes my sisters set their alarm clocks to remind me to take my medication. Its only my immediate family who knows that l am HIV positive (Bigboy, 16)*.

From these responses the importance of family support is emphasized. Unfortunately for some stigma was both experienced and anticipated from their family members. Because a seropositive status in children often represents HIV positive parents and siblings, family members might feel the need to support and maintain their family status secret.

Participants were asked about their experiences with stigma. Due to the fear of being stigmatized, ABYM living with HIV have developed coping strategies to help them navigate their lives and avoid stigma. Adolescents conveyed a sense that they learned to live with their diagnosis and that they are getting on with their lives.

*I go to the clinic when they are about to close. That way my chances of meeting people l know are slim. I first check from a distance to see if there are people l know around. If there are, l walk around the clinic until they are gone then l collect my medicine. (Jedza, 17 years)*

*I just go in anytime of the day. If l meet someone l know l just tell them l am collecting my granny's BP tablets. Everyone knows that my granny takes BP medication. (Tiko, 20 years)*

These sentiments reveal the lengths ABYM go to just to protect and maintain their secrecy. Secrecy was important rather than risking accidental disclosure to the community at large.

#### Treatment Adherence

Overall treatment adherence was poor with 19 ABYM reporting partial adherence to medication. When asked about their medication regimen, one boy reported taking their medication religiously and one reported that he stopped taking medication voluntarily although he still attended social support services and collects his medication and throws it away.

*Sometimes I forget because I will be playing with other guys. Or sometimes I will take my medication but I will take them late. (Tinotenda, 15)*

*I also skipped my medication because they make me feel lightheaded. They make me sick. When l am writing exams, l stop taking them then resume after exams. (Talent, 15 years)*

*I have stopped taking medication. I can't be bothered. I collect from the clinic because l don't want my mother to bother me but l throw the pills away. I flush them in the toilet. (James, 20)*

The response highlighted important issues such as pill burden which are useful for ABYM to improve on treatment adherence. Hunger and taking medication adherence for granted proved to influence how ABYM took their medication. Taking medication on an empty stomach and the side effects made the pill burden heavy to bear and led to medication default.

*Food is a problem in my household. I stay with my aunt and she doesn't earn much. She had to produce my parents' death certificate for her to be considered for food distribution by a local Non-Governmental Organization (NGO). But the food is not enough because we are a big family. Maybe if we can get enough food it will be better. (Talent, 15 years)*

ABYM also highlighted what they felt needed to be changed to improve medication adherence;

*I feel they need to locate the dispensary at the back of the hospital. Sometimes l default on collecting medication because there will be too many people at the clinic and l can't risk being seen. Again, these green files they give us makes us easily identifiable. Everyone knows there are for ART patients and its embarrassing. (Tiko, 20 years)*

*Sometimes l don't get transport money to go to the clinic to collect medication. I can't walk the long distance. So, you see, l don't default by choice. It's because l don't have transport money. (Taita, 19 years)*

This highlights how HIV interventions need to be context specific and consider the protection and secrecy of ABYM to avoid accidental disclosure.

## Discussion

The study revealed that the fundamental challenge that parents and caregivers of HIV-infected adolescents face is the initial disclosure of HIV seropositive status to their children (26). A greater proportion of the respondents (*n* = 14) of ABYM had not been told of their HIV diagnosis by their caregivers initially, but they were told by HIV counselors. The probable reason may be because parents and caregivers want to protect their children from the burden of HIV until the time they can understand the implications of their status (5). Majority of ABYM (*n* = 11) had their status disclosed to them at age ranging from 10 to 15 years. This is the age where adolescents begin to get an idea and grasp of the direction their lives are taking and the stage at which maturation begins. Participants reported that there were either sick and in hospital when their status was disclosed to them or they took it upon themselves to find out their status and got tested. This was consistent with other studies which also produced the same results (27). Not only do caregivers wait till the children have matured to disclose to them, but also keep postponing it until the child get tested on their own initiative or until the child begins to question the reason why they take medication everyday even when they don't feel sick (26, 28). This implies that parents and caregivers postponed disclosure until the benefits outweighed possible risk to their children. The benefits included understanding and acceptance of the implications of the disease as well as the ability to manage the disease for positive health outcomes. Several studies also produced the same findings (29, 30). However, the study is in direct contrast to recommendations that suggest that disclosure should be the key responsibility of the primary caregiver with the help and support of healthcare workers (31, 32). This confirms the complexity of disclosure and underscores the need to consider the process as delicate.

Majority of respondents did not receive the news of their HIV seropositive status well. Ten reported feelings of anger, disbelief and stress as well as feeling betrayed by their parents. Four were suicidal and isolated themselves from people for some time, three accepted their status and four did not know how to react because they were too young to understand about HIV. This is due to widespread perceptions that HIV is an incurable and fatal disease. However, disclosure helped them to understand more about HIV including the process of taking medication and self-care in the quest for an improved quality of life. Previous studies in Zimbabwe and South Africa also testified to the fact that adolescents reported feelings of shock and betrayal soon after having their HIV-positive status disclosed. Acceptance later followed in the understanding that knowing one's own HIV status is important for treatment adherence and better quality of life (33, 34). This implies that HIV positive ABYM have the ability to adapt to and accept their status once the initial anger period has passed true to the adage that time heals everything.

As mentioned in the preceding discussion, the decision for HIV disclosure was a matter of assessing and calculating perceived benefits and risks related to the disclosure. Stigma and rejection were recurrent in the lives of HIV positive ABYM from initial disclosure to onward disclosure. This testifies to how HIV/AIDS has remained a stigmatized disease even after years of information and knowledge shedding more light on how the disease is acquired, managed and avoided. This is worrisome for people living with HIV infection and policy makers because this has negative impacts on the efforts and work of researchers as well as the quality of life for people living with HIV. As Bernado, suggests, the fear of stigma impacts on disclosure, treatment, and social relations (35). The view that HIV status was one's secret and they were not obliged to tell other people was unanimous among study participants. Selective disclosure was the norm with 8 ABYM not disclosing their status to anyone, four disclosing to their families, three to their classmates and two to their teachers. Control over who they disclose to allows them to control stigma. As most adolescents desired to live a normal healthy life, they perceived onward self-disclosure negatively and felt that they would be treated differently if they disclosed their HIV status to friends and romantic partners. Consequently, most maintained secrecy in order to be accepted by their peers. In their context, peer acceptance was much more important than disclosure of their HIV status, which most felt had nothing to do with their friends. Maintaining secrecy was also a way to protect themselves from stigma and isolation from others within the home and school environment (36).

However, in contrast with previous studies, disclosure and acceptance of HIV status did not mean that they adhered to their medication. Previous studies proved that once their HIV seropositive status had been communicated to them and they had accepted their status, HIV positive adolescents felt motivated to adhere to their medication (36). Evidence points to the fact that adherence is challenging in children. Only one boy reported taking his medication religiously. Nineteen took their medicine occasionally either because they forget or because they don't want people to see them taking medication. One boy had completely stopped because he did not see the reason to continue taking them. This is supported by studies in Uganda where it was found that adolescents had problems with adherence (37, 38). Reasons for non-adherence were varied. Poverty emerged as a major barrier to art adherence. ABYM in the study highlighted that they sometimes fail to go to the clinic to collect medication because they have no transport money and even if they manage to collect medication they sometimes skip doses because they will be feeling week due to hunger and if they take medication on an empty stomach they get sick. Weiser et al. yielded the same results in Uganda where food insecurity and poverty prevented total adherence to ART (38). As such, poverty and hunger imposed an additional burden to the already overburdened adolescents.

Pill burden or treatment fatigue emerged as reason for non-adherence. All of the study participants mentioned that in the beginning stages of ART they felt dizzy and sick after taking ART and stopped taking medication because of that. Non-adherence leads to drug resistance and opportunistic infections soaring therefore compromising the quality of life. Pill burden takes different dimensions that may present as quantity of medication one takes, the taste and the challenge of remembering to take pills every time. ABYM reported that they did not want everyone to know that they took pills. Not everyone wanted their status known to peers since disclosure posed both positive and negative outcomes. Evidence from the study reveals a boy who had his family members separate his utensils with them and would not share anything with him including bath soap. Another boy had his classmates shun him and separate from him after disclosure. For some the disclosure experience was pleasant as it led to their teachers and family members understanding them and even reminding them to take their medication.

Lack of adolescent friendly HIV clinics was also a major obstacle to adherence. ABYM mentioned that there were given green cards that identified them as HIV patients and set them apart from the other patients. Even the location of the dispensary was not strategic. Adolescents felt that the dispensary entrance should have been located at the back rather than at an open area where everyone could see them collecting medicine. These barriers have been reported previously in other studies and are not unique to adolescents (39).

## Limitations of Study

This study has several limitations. The study design was conducted in one rural hospital in Zimbabwe in a small sample of ABYM. As such, study results cannot be generalized and are only particular to the study area. Again, respondents were recruited at their regular clinical reviews limiting the generalizability of the findings to only those who are compliant leaving those who are not compliant with care instructions or had no access to care. Those adolescents may have different views or different impacts of disclosure. Furthermore, the study relied on self-reported narrative which compromises the quality of the findings, therefore a quantitative study might be appropriate to enhance reliability and replicability.

## Implications for Future Research

Despite the limitations above, our study findings have significant implications for practice.

As ABYM living with HIV continue to benefit from increased access to ARVs, there is an urgent need to create safer, more supportive environments for them so that they do not just live longer but that they may enjoy happy, fulfilled childhoods in which they feel protected, valued and loved. This can be through community involvement working alongside public health and other systems which is essential in advocating for a robust response to the epidemic, delivering services that can reach everyone in need and tackling HIV-related stigma and discrimination. The Zvandiri Programme in Zimbabwe has taken the initiative by training HIV positive adolescents to deliver a range of counseling, training and advocacy activities which seek to reduce stigma toward children with HIV and to help children to develop the confidence and skills to cope with stigma (40). The programme also emphasizes the importance of peer support to support HIV positive youths. WHO recommends the view that peer support is more responsive, accepted, and relevant in encouraging adolescents (10–19) to seek and remain engaged in HIV care (41). The local Zimbabwean Friendship Bench (FB) model, has also proved to be the best fit locally in reducing suicidal ideation and improves adherence to PLWHIV findings that are congruent with other studies (42, 43). Detailed description of FB intervention model can be found elsewhere (44).

The study has emphasized the need for a combination approach to HIV interventions rather than a single approach. HIV is as a result of biological, behavioral, social and structural factors. Not only do we need multilevel approaches to HIV interventions, but we also need to integrate them to ensure they are mutually supportive and reinforcing (45). Future interventions to improve adherence should address psychosocial factors such as treatment fatigue, depressive symptoms, disclosure and family and household dynamics amongst adolescents. Integrating psycho-social interventions with bio-medical/educational/behavioral/structural interventions enhance ABYM knowledge and skills and motivates them to adapt healthy lifestyles. Financial cash flows can greatly build resilience of adolescents by providing financial muscle hence enabling them to travel to hospitals for regular medical check-ups, food security and much needed instrumental social support (46). Financial support also reduces risky behaviors such as transactional sex and drug abuse.

In addition, there is need for specific adolescent friendly clinics to prevent in-advent disclosure. Literature recommends the integration of youth-friendly services such as evening clinics, adolescent clinic days (not just HIV positive adolescents) and youth-friendly waiting areas within ART programmes (47).

To reduce pill burden, new technologies are needed to reduce the number of pills required both in dosage and frequency. Long-acting injectable ART medications may reduce the daily pill taking responsibility if they are taken for example, every quarter year (48). However, such treatment advances require time before they are successfully implemented for adolescents and youth, given the challenges associated with testing the safety and efficacy of biomedical strategies in younger populations.

Finally, the issues related to initial and onward disclosure of a HIV seropositive status (highly stigmatized) in adolescence highlight the importance of expanding public health programs and policies to adopt solid strategies that help youth navigate these complex social situations. Adolescents, being in their formative years, struggle to disclose their HIV status to their partners and therefore need specific skills to help them cope with these new and often complicated social and sexual dynamics. Careful attention is needed to address the special barriers faced by HIV+ adolescents and youth to support them in their transition to adulthood.

## Conclusion

The study has testified to the efficacy of a socioecological approach to acknowledge broader social factors into the decision-making of both ABYM and in parental HIV disclosure as well as the coping strategies and treatment adherence journeys of ABYM. The Socioecological model acknowledges ABYM interaction and reliance on broader environmental systems and stresses the importance of interactions between individuals and environment that influence behaviors and health outcomes. The pervasive fear of stigma influenced and shaped ABYM's adherence behavior. Fear of stigma affected the time and place to take medication, the visit to the clinic for ART refill, and self-disclosure of HIV status. There is need to encourage adolescents to self-disclose their HIV status to friends since the fear of unintended disclosure fueled perceived stigma. Interventions for ABYM should acknowledge psychosocial factors including the dynamics of disclosure, treatment adherence as well as onward disclosure to. Family and household dynamics should also be taken into consideration. The results have various implications with relevance to the level of care that HIV infected adolescents need. Most important is the fact that for HIV adolescents to have a better quality of life, there is need to provide a comprehensive and multi-sectorial programme that addresses their various challenges, many of which are not medical.

## Data Availability Statement

The raw data supporting the conclusions of this article will be made available by the authors, without undue reservation.

## Ethics Statement

The studies involving human participants were reviewed and approved by Medical Research Council of Zimbabwe approval number (MRCZ/B1794) and University of KwaZulu Natal BREC approval number (BE475/19). Written informed consent to participate in this study was provided by the participants' legal guardian/next of kin.

## Author Contributions

RK contributed to the research through conceptualizing the research protocol, data analysis, and final write-up. CJ contributed to the research by collecting data and part of the write-up. KG supervised and edited the final article. All authors contributed to the article and approved the submitted version.

## Conflict of Interest

The authors declare that the research was conducted in the absence of any commercial or financial relationships that could be construed as a potential conflict of interest.

## Publisher's Note

All claims expressed in this article are solely those of the authors and do not necessarily represent those of their affiliated organizations, or those of the publisher, the editors and the reviewers. Any product that may be evaluated in this article, or claim that may be made by its manufacturer, is not guaranteed or endorsed by the publisher.
